# Mechanical and Microstructural Investigations of AA2124/SiC Metal Matrix Composites After Creep

**DOI:** 10.3390/ma18194495

**Published:** 2025-09-27

**Authors:** Agnieszka Rutecka, Katarzyna Makowska, Zbigniew Ludwik Kowalewski

**Affiliations:** 1 Faculty of Civil Engineering, Warsaw University of Technology, Al. Armii Ludowej 16, 00-637 Warsaw, Poland; agnieszka.rutecka@pw.edu.pl; 2 Faculty of Mechatronics, Armament and Aerospace, Military University of Technology, gen. Sylwestra Kaliskiego 2, 00-908 Warsaw, Poland; 3 Institute of Fundamental Technological Research (IPPT PAN), Pawińskiego 5B, 02-106 Warsaw, Poland; zkowalew@ippt.pan.pl

**Keywords:** creep, damage, deformation history, metal matrix composites (MMCs), microstructures

## Abstract

The AA2124 aluminium alloy-based metal matrix composites (MMCs) reinforced with the silicon carbide (SiC) were examined under tensile creep at 300 °C. The tests were carried out for the materials of different SiC particle size (3 µm and 0.6 µm) and amount (17 vol.% and 25 vol.%). Creep curves under different constant stresses are presented. A high stress sensitivity of the composites tested was identified for a very narrow range of stress values. As a result, a threshold stress range separating the slow and fast creep stages was easily identified at around 5 Mpa for the composite with a larger SiC particle size and lower content and around 1 Mpa for the two other composites. It means that a very small change in stress applied to the structural element at elevated temperatures may lead to its very rapid collapse or even the destruction of the whole structure. The experimental programme was supplemented by the microstructural observations carried out using the scanning electron microscopy providing data necessary for better understanding the damage mechanisms of the material subjected to creep. An influence of voids on the mechanical response and fracture zones was identified. Attention was paid to the nature of degradation of the composites.

## 1. Introduction

Nanocomposite materials are valued for their exceptional mechanical properties in terms of strength and lightness [1,2]. Nanometric reinforcements can significantly reduce low fracture toughness, low ductility and poor machinability [3]. Modification of nanocomposites includes changing the type, size and concentration of reinforcement as well as changing the type of matrix [4,5]. The formation of MMCs has attracted great interest due to its excellent combination of strength, stiffness and wear resistance [6,7,8,9,10]. They also draw attention for their low density [4]. Moreover, MMCs combine the strength and rigidity of non-metallic reinforcing particles with the ductility of a metal matrix [11]. The Hubble telescope is produced from aluminium and graphite fibres [12]. These composite parts are very durable and lightweight [12]. Nickel-based metal matrix nanocomposites can be composed of carbon nanotubes and SiC particles [3]. Nickel has greater mechanical strength and a higher sintering temperature compared to aluminium. Carbon nanotubes are added to the nickel matrix because nickel has a low tendency to form carbides [13]. Using SiC particles and a Ni matrix, Nikasil can be produced, which is used in the manufacture of cylinders and piston rings [14]. Moreover, it was applied in racing engines of Formula (1) [15,16].

Graphene was considered as a reinforcement in copper metal composites, but unfortunately, the bonding between copper and graphene is weak. The molecular-level mixing process was attempted to achieve a stronger interface bonding between the reinforcement and the matrix [17]. Ensuring the correct bonding of two different materials is crucial in order to obtain the desired operational properties [18].

Titanium nanocomposites with TiO_2_ nanoparticles have better tribological properties than pure titanium alloys [19]. Titanium alloys are characterised by high structural strength and very good corrosion resistance. On the other hand, they have relatively low hardness and insufficient tribological properties [20]. The addition of TiO_2_ to the engine oil reduced friction and wear [21]. Graphene also improves the tribological properties of magnesium alloys [3]. Simultaneously, aluminium oxide reduces the wear rate and friction coefficient of the silver-based matrix.

This work focuses on aluminium matrix composites and nanocomposites reinforced with SiC. In [22] fracture toughness of an aluminium die-cast alloy (ADC-12) and 10% weight SiC particles for reinforcement was assessed as promising. Authors of [23] focused on tribological analysis of an aluminium matrix composite reinforced with SiC and Mg. Despite the above-mentioned achievements, due to the lack of sufficient experimental data necessary for a more complete understanding of the behaviour of MMCs under constant load, the creep properties of selected composites of this type were investigated in this article.

Creep is usually of concern to engineers and metallurgists when evaluating components operating under high stresses or elevated temperatures [24]. The interest in creep of MMCs is mainly motivated by their improved specific stiffness, strength and wear resistance as compared to the unreinforced matrix. The most commonly used metal matrices for MMCs are based on aluminium and titanium. One major motivation for the usage of MMCs based on Al or Mg is their superior mechanical properties at elevated temperatures compared to the matrix alloys. The reinforcing phase of a MMC has a higher creep resistance than the matrix at a given temperature. Potentially, this can improve creep properties of the MMC as compared to the unreinforced matrix.

Tensile creep tests on aluminium-based composites reinforced with SiC particles of different sizes showed that their creep rate for small particles (3.5 µm) was two–three orders of magnitude lower than that of the pure aluminium [25]. However, the creep resistance of composites reinforced with large SiC particles (10 µm and 20 µm) was essentially identical to that of the pure aluminium. In a further study, Whitehouse and Winand [26] systematically tested the creep response of powder-route aluminium reinforced with different sizes, shapes and volume fractions of SiC. The creep resistance was found to increase with the increasing aspect ratio. Whitehouse et al. [27] similarly observed that the creep resistance of composites containing fine whiskers of 1 µm diameter was better than that of composites containing larger whiskers of 3 µm diameter. A comparison between the creep characteristics of squeeze-cast AZ91 and QE22 magnesium alloys reinforced by 20% Al_2_O_3_ short fibres and unreinforced matrix alloys showed that the creep resistance of the reinforced materials is considerably improved compared to the monolithic alloys [28,29]. Microstructural investigations by TEM revealed good fibre/matrix interface bonding during creep exposure, and creep strengthening was attributed to effective load transfer between the matrix and fibres. By contrast, no substantial increase in creep strength was observed if particles were used as reinforcements in QE22 [30]. In Winand et al.’s study [31], it was shown that when using neutron diffraction, the reinforcement often bears the larger share of the applied creep stress, and thus, improves the creep resistance of the composite. The features of the fibre/matrix interface are obviously important for the effective load transfer and creep behaviour. This was also confirmed in the study by Li et al. [32]. The mechanical properties of the metal matrix can have a strong influence on the creep behaviour of the composite, particularly in the tertiary creep stage. This was demonstrated by Lee and Yu [33]. Some other issues related to creep behaviour of MMCs are discussed in the relatively rich available literature [34,35,36,37,38,39] Despite many investigations carried out on metal matrix composites reinforced by aluminium particles, there are still many issues that require more thorough studies. Among the most important mechanical effects, one can indicate the existence of a proper steady state in the composite materials [40]. In [41] it was pointed out that a proper steady-state region exists only for the Al-6061 based MMC. Contrary to that case, for the Al-2024 composite the steady-state region was not found [41]. Barth et al. [42] emphasised that a well-defined steady-state creep exists only below a certain stress called the threshold stress. The threshold stress of Al-2024 reinforced by 15% SiC was analysed in [43]. It was found that the magnitude of the threshold stress depends on both the level of applied stress and testing temperature.

The Al-2124 ± 10% SiC (particles) were tested by Li and Langdon [44]. For materials like metal matrix composites, logarithmic plots of strain rate against applied stress exhibit very high values of apparent stress exponent n at the lowest levels of the applied stress. That is why effective stress is calculated in such materials as applied stress minus threshold stress.

The creep behaviour of the 2124 Al-alloy unreinforced and reinforced with 25% of SiC particles of different sizes was investigated by Requena et al. [45]. A low-stress region with the Norton’s creep exponent n around 3 and high-stress region with n increased to values greater than 10 were observed. Two effects that can contribute to the threshold stress presence in Al-based metal matrix composites were recently proposed by Fernández and González Doncel [46]. One of them is a reduction in the stress within the matrix due to load transfer from the matrix to reinforcement. The second one is the threshold stress as a result of dispersoids presence.

The 20% SiC 2124 Al composite was tested by Kim et al. [47]. The threshold stress that was evaluated allowed the correct n value to represent the exact behaviour of the Al-based metal matrix composite. The 2124Al alloy and two 2124Al-based composites reinforced with 20% of particulate SiC (3.5 mm) and SiC whiskers, respectively, were investigated by Ma and Tjong. [48]. At the beginning the threshold stress for the particulate composite and the whisker-reinforced threshold stress increased with temperature, and subsequently, at higher temperatures, became lower.

The 2124 Al alloy reinforced by 20% silicon carbide particulates subjected to creep shear stresses was investigated by Cadek et al. [49]. The threshold stress decreased linearly with increasing temperature. The 2124 aluminium composite reinforced by 5% silicon carbide particulate was tested by Lin et al. [50]. The results showed that the origin of the anomalous stress dependence of the creep rate in the composite and unreinforced matrix alloy is due to the threshold stress which depends strongly on temperature. The creep behaviour of 2124Al reinforced with 10–30% of SiC particles was investigated by Ryu et al. [51]. It was observed that the minimum creep rate of the composites decreased with increasing the volume fraction of SiC particles.

Instead of threshold stress as the parameter for which strain rate is equal to zero, the experimental results captured in this work enable the identification of a threshold stress range, as the stress limits which separate a slow and fast creep development ranges in the composites were tested.

The dynamic creep process of the 7055 Al alloy with reinforcement containing Al_2_O_3_, ZrB_2_ and Al_3_(Er, Zr) was analysed in [52]. It was found that the dominative creep mechanism in the 7055 Al/Al_2_O_3_/ZrB_2_/Al_3_(Er, Zr) composite was dislocation climbing and the true stress exponent was five. The A356 aluminium alloy with a reinforcement of 0.2%, 0.5% and 1 wt.% multi-walled carbon nanotubes (MWCNTs) was tested in [53]. The introduction of 0.2% nanotubes reduced creep failure strain by 13%. Moreover, the minimum creep rates of A356/0.2 MWCNTs, A356/0.5 MWCNTs and A356/0.5 MWCNTs increased by 0.46%, and decreased by 32% and 3.2%, respectively. Unfortunately, the authors did not investigate damage mechanisms. In [54] (ZrB_2_ + Al_2_O_3_)_np_/7055 Al under various applied stresses was tested. It was observed that the steady-state creep rates of the composites with a volume fraction of 3% were 9.7 to 22.9 times lower than those of the pure alloy. It was also found that the creep rate of the 7055 Al alloy and composite material is controlled by dislocation climbing for temperature in the range of 473–573 K and for stress between 60 Mpa and 80 Mpa [54].

The novelties of this paper are represented by new creep data for the AA2124 aluminium alloy reinforced by SiC ceramic particles of different sizes and amounts. Such kind of composites can be commercially used in aerospace and automotive industries as well as space and defence optical systems.

The aim of this paper was to examine how the grain size of reinforcement and its percentage content affect the properties of the specific creep of the composites tested. Since composite materials are more and more commonly used in aerospace and automotive industries, it seems to be reasonable to investigate their properties, especially at elevated temperatures. However, the most important aspect of the study is the evaluation of how changes in creep stress affect the creep behaviour of aluminium alloy-based nanocomposites.

## 2. Materials and Methods

Tensile creep tests were performed for three types of metal matrix composites. The AA2124 matrix was reinforced by SiC (silicon-carbide) ceramic particles. The amount of SiC particles was equal to 17 vol.% and 25 vol.%. The average size of SiC particles was equal to about 3 µm (coarse type) and 0.6 µm (fine type). During the MMCs’ production, metal powders and SiC particles were subjected to high energy mixing, hot isostatic compaction, forging and T6 CWQ (Cold Water Quenching) heat treatment. The specimens were manufactured using machine cutting and then polished. More information about the materials can be found in [55,56]. The composites with a certain amount and size of SiC were chosen to be investigated as those to be commercially used in the automotive and aerospace industries.

The following materials were tested:

AA2124 with 17 vol.% of SiC particles of average size equal to 3 µm;

AA2124 with 17 vol.% of SiC particles of average size equal to 0.6 µm;

AA2124 with 25 vol.% of SiC particles of average size equal to 0.6 µm.

**Figure 1:**
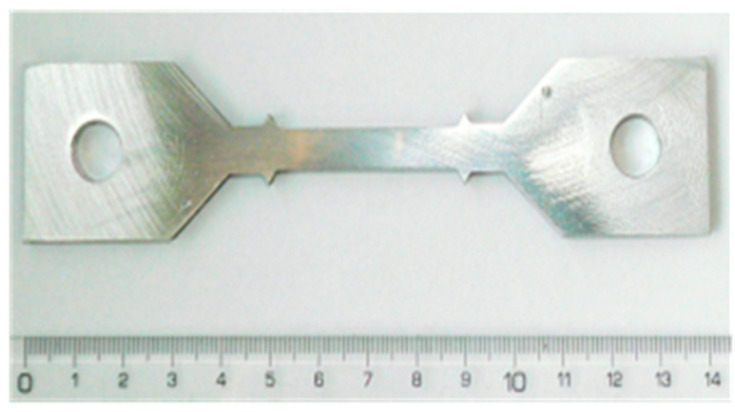
Creep specimen.

Flat specimens with rectangular cross sections manufactured from the materials were subjected to creep (Figure 1).

Creep tests were performed on the typical creep testing machines. The specimens’ cross-section dimensions and gauge length were equal to 7 mm × 5 mm and 40 mm, respectively. The specimen geometry is shown in Figure 1 in [57]. A mechanical extensometer was mounted on specimens and axial strain was measured as a function of time. A furnace mounted on the creep testing machine allowed us to keep a constant high temperature along the specimen. Specimens subjected to the given levels of temperature were loaded to obtain step-increased tensile stress conditions for the specified time period or to keep the constant load during the whole experiment. After preliminary tests, the temperature of 300 °C was chosen to execute the subsequent creep tests. Creep parameters are shown In Table 1, Table 2 and Table 3.

Fracture analysis of selected specimens after creep was performed using a JEOL 6360 LA scanning electron microscope (JEOL Ltd., Tokyo, Japan). Next, the fractures and specimens from interrupted creep tests were cut and embedded in resin parallel to the loading axis, then ground down to half their thickness in order to observe material degradation after the load tests. A quantitative analysis of defects in the creep-tested specimens was performed using representative images. The following parameters were determined using the planimetric method:

A_A_ = V_V_ [%]—volume fraction of the voids;

F_śr_ [μm]—mean Feret diameter of the void;

N [1/μm^2^]—void density in the specimen after creep;

P [1/μm^2^]—particle fragmentation amount.

## 3. Results and Discussion

### 3.1. Creep Tests on AA2124 + 17 vol.% SiC (3 µm) Composite

**Figure 2:**
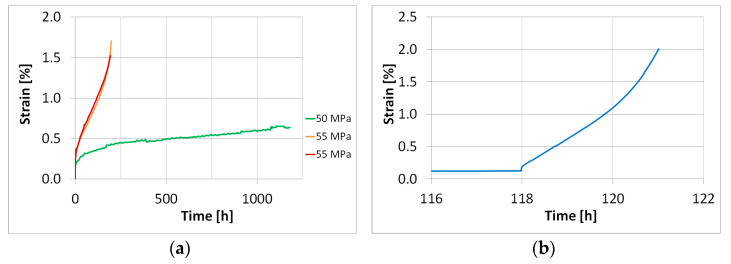
Comparison of creep curves for different values of stress for AA2124 + 17 vol.% SiC (3 µm): specimens 2 and 3 (stress 55 MPa) to the curve obtained for specimen 1 (test stopped) (stress 50 MPa) (**a**); creep curve for specimen 4 under stress of 39 MPa, and subsequently at 60 MPa, and finally stopped (**b**).

As it was mentioned earlier, the tests were performed at 300 °C for all composites in question. The nominal tensile stress 50 Mpa was applied for the creep of the specimen 1, Figure 2a. The test did not reach tertiary creep during 1180 h of loading and it was stopped for the strain equal to 0.65%.

Simultaneously, specimen 2 was subjected to a nominal applied stress of 55 Mpa. The test was stopped after 195 h for the strain equal to 1.5% at the beginning of tertiary creep; therefore, it was possible to evaluate the minimum creep rate and creep rate at the beginning of tertiary creep. Specimen 3 (Figure 2a) was tested under the same conditions as the specimen 2. Also, in this case both creep rates were determined successfully. The test was stopped after 197 h for the strain equal to 1.7%.

A comparison of creep curves for two specimens tested in the same conditions subjected to nominal stress of 55 Mpa shows a good agreement, Figure 2a, thus provides proof of the high accuracy of the testing technique. The specimen tested under the nominal applied stress of 50 Mpa exhibited smaller strain values than those obtained at the creep under stress of 55 Mpa. It has to be emphasised that the creep process did not reach the tertiary period in this case.

In the next step of the experimental programme, specimen 4 was subjected to two subsequent stress levels, 39 MPa and 60 MPa. One can easily observe that for the higher stress (60 MPa) the material showed a very quick start into the tertiary creep, Figure 2b. The details of creep tests are shown in Table 1.

### 3.2. Creep Tests on AA2124 +17 vol.% SiC (0.6 µm)

**Figure 3:**
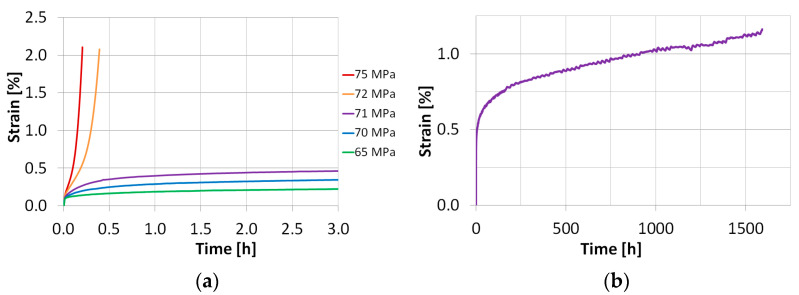
Comparison of creep curves for different values of stress for AA2124 + 17 vol.% SiC (0.6 µm) (**a**); creep curve for specimen 7 tested under a stress of 71 MPa (**b**).

Creep tests for the AA2124 + 17 vol.% SiC composite (0.6 µm) were carried out at 300 °C for five stress levels. The creep curves for stresses equal to 65 MPa, 70 MPa, 71 MPa, 72 MPa and 75 MPa are compared in Figure 3a. Specimens 5 and 6 tested at nominal applied stresses of 65 MPa and 70 MPa, respectively, did not reach the tertiary creep, Figure 3a in the planned tests duration. In the case of specimen 7, the stress was increased by 1 MPa to obtain 71 MPa (Figure 3a,b). Despite that, the tertiary creep was not achieved again.

Specimen 8 was subjected to stress 1 MPa higher than that applied to specimen 7, giving the nominal stress of 72 MPa. As it is clearly seen, the tertiary creep was achieved immediately in this case, and after 0.4 h the test was stopped at the strain of 2%. Subsequently, a single creep test on specimens 9 and 10 was carried out under a nominal applied stress of 75 MPa.

One can indicate a high creep strain sensitivity of the material within the very narrow stress range. A difference equal to 1 MPa led to the substantial variation in the creep course (see the curves carried out under nominal applied stress 71 MPa and 72 MPa); thus, defining a threshold stress range separating slow and fast variants of the creep process.

One can conclude that the AA2124 + 17 vol.% SiC with finer particle reinforcement (0.6 µm) is more creep strain resistant compared to the same composite of coarser (3 µm) SiC particles. It was confirmed by the higher values of stress that were used in the tests of this composite. The details of the creep tests are shown in Table 2.

### 3.3. Creep Tests on AA2124 + 25 vol.% SiC (0.6 µm)

**Figure 4:**
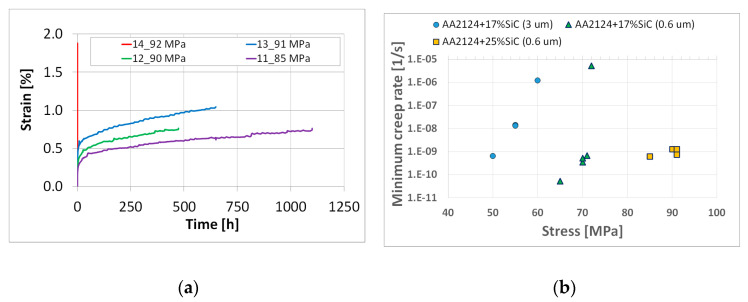
Comparison of creep curves for specimens 11, 12, 13 and 14 made of the AA2124 + 25 vol.% SiC (0.6 µm) for a range of stress levels (**a**); minimum creep rate vs. nominal applied stress (**b**).

In the next step of the experimental programme, the specimens of composites with a higher amount of SiC (25 vol.%) and finer grains of reinforcement (0.6 µm) were subjected to creep tests at 300 °C (Figure 4a).

Specimens 11, 12 and 13 tested at 85 MPa, 90 Mpa and 91 Mpa, respectively, exhibited a different stress sensitivity, Figure 4a. Although none of them reached the tertiary creep stage, they ruptured during the secondary creep stage. In order to find existence of the threshold stress range of this material, specimen 14 was subjected to creep under a nominal applied stress of 92 Mpa, which was slightly higher than the last one considered in this part of the experimental programme. It is easy to see that the material exhibited a very high strain rate. To identify possible features of this effect, the test was stopped for further inspection after 0.096 h when the strain attained a value of 1.89%.

The AA2124 + 25 vol.% SiC (0.6 µm), similarly to the AA2124 + 17 vol.% SiC (0.6 µm), exhibited a very narrow (1 MPa) range of stress values that separated slow and fast variants of the creep process. The details of the creep tests are shown in Table 3. The minimum creep rates vs. nominal applied stress for the materials tested are presented in Figure 4b. The minimum creep rates for AA2124 + 17 vol.% SiC (3 µm) were in the range from 6.5 × 10^−10^ s^−1^ to 1.2 × 10^−6^ s^−1^ with the highest value for very fast creep equal to 1.2 × 10^−6^ s^−1^. The minimum creep rates for AA2124 + 17 vol.% SiC (0.6 µm) were in the range from 5.3 × 10^−11^ s^−1^ to 5.3 × 10^−6^ s^−1^ with the highest value for very fast creep equal to 5.3 × 10^−6^ s^−1^. The minimum creep rates for AA2124 + 25 vol.% SiC (0.6 µm) were in the range from 6.4 × 10^−10^ s^−1^ to 1.3 × 10^−9^ s^−1^, but the highest value for very fast creep was difficult to evaluate.

### 3.4. Microstructural Observations

**Figure 5:**
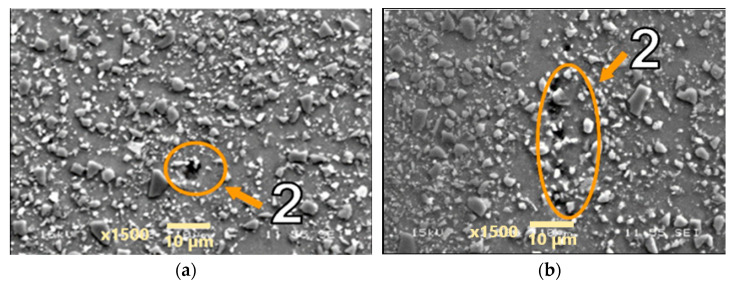
Microstructural effects due to creep of the AA2124 + 17 vol.% SiC (3 µm)—specimen 1 tested at 300 °C under stress of 50 MPa—longitudinal cross section: (1) areas without SiC particles, (2) voids. (**a**,**b**) show different distribution of defects on the same sample.

**Figure 6:**
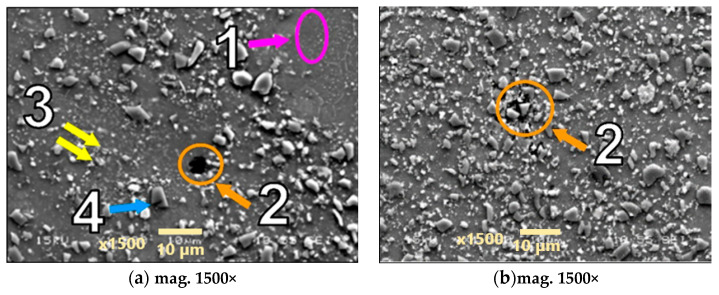
Microstructural effects due to creep of the AA2124 + 17 vol.% SiC (3 µm)—specimen 3 tested at 300 °C under stress 55 MPa—longitudinal cross section: (1) areas without the SiC particles, (2) voids, (3) defragmentation of SiC particles, (4) cracks of SiC particles.

**Figure 7:**
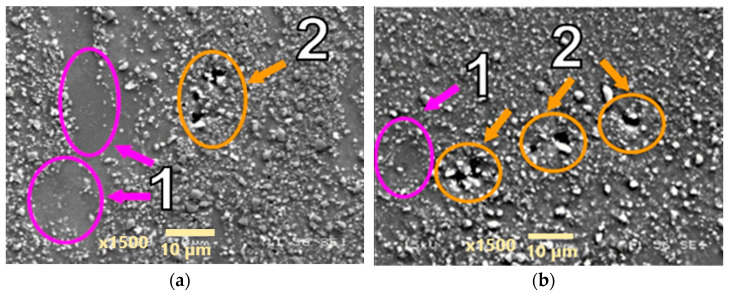
Microstructural effects due to creep of the AA2124 + 17 vol.% SiC (0.6 µm)—specimen 6 tested at 300 °C under stress of 70 MPa—longitudinal cross section: (1) areas without SiC particles, (2) voids. (**a**,**b**) show different distribution of defects on the same sample.

**Figure 8:**
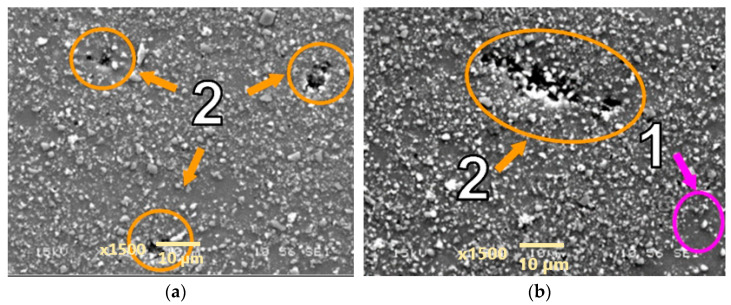
Microstructural effects due to creep of the AA2124 + 17 vol.% SiC (0.6 µm)—specimen 7 tested at 300 °C under stress of 71 MPa—longitudinal cross section: (1) areas without SiC particles, (2) void. (**a**,**b**) show different distribution of defects on the same sample.

**Figure 9:**
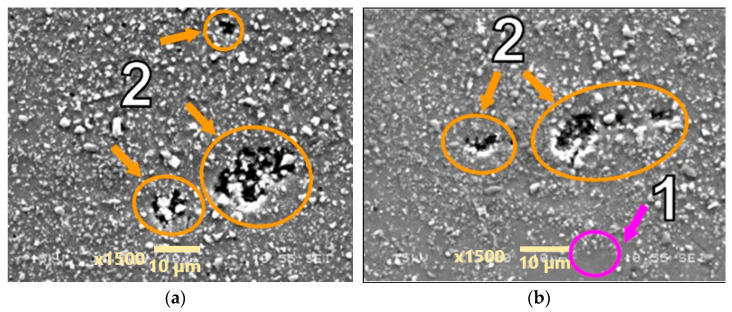
Microstructural effects due to creep of the AA2124 + 17 vol.% SiC (0.6 µm)—specimen 6a tested at 300 °C, under stress of 70 Mpa—longitudinal cross section: (1) areas without the SiC particles, (2) voids. (**a**,**b**) show different distribution of defects on the same sample.

**Figure 10:**
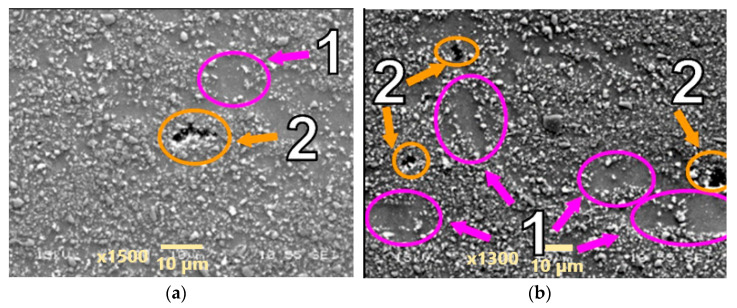
Microstructural effects due to creep of the AA2124 + 25 vol.% SiC (0.6 µm)—specimen 4 tested at 300 °C, under stress of 90 MPa—longitudinal cross section: (1) areas without the SiC particles, (2) voids. (**a**,**b**) show different distribution of defects on the same sample.

**Figure 11:**
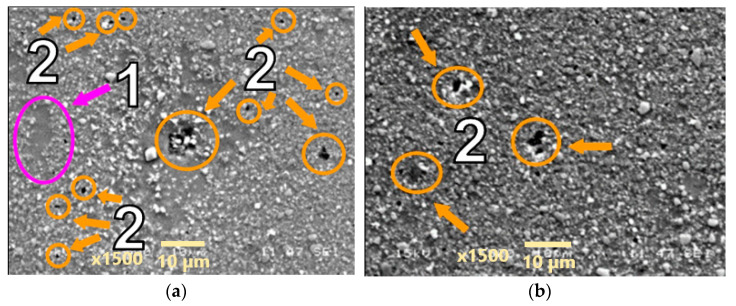
Microstructural effects due to creep of the AA2124 + 25 vol.% SiC (0.6 µm)—specimen 13 tested at 300 °C under stress of 91 MPa—longitudinal cross section: (1) areas without SiC particles, (2) voids. (**a**,**b**) show different distribution of defects on the same sample.

Microstructures of the materials after creep are presented in Figure 5, Figure 6, Figure 7, Figure 8, Figure 9, Figure 10 and Figure 11. Scanning electron microscope observations have shown that the reinforcement is generally evenly distributed in all types of the composites tested, i.e., AA2124 + 17 vol.% SiC with the reinforcing particles of 3 µm; AA2124 + 17 vol.% SiC with the reinforcing particles of 0.6 µm and AA2124 + 25 vol.% SiC with the reinforcing particles of 0.6 µm. Randomly distributed small areas without SiC particles were observed only locally in the materials tested. Those regions exhibited both an isotropic and anisotropic character. Creep loading of the AA2124 + 17 vol.% SiC with the reinforcing particles of 3 µm under the stress of 50 MPa led to some voids being created in the material. Such defects appear singly or side by side, Figure 5. An increase in the creep stress to 55 Mpa led to a significant increase in the creep rate and to the formation of similar defects, Figure 6. Single voids formed in the matrix with locally less frequently distributed reinforcement have more favourable conditions to growth and reach larger sizes, Figure 6a. In areas with locally more densely distributed reinforcement, the voids could not change their size, because their growth was limited by SiC particles, Figure 6b. At the stress of 55 Mpa, a defragmentation of the reinforcing particles of 3 µm was also observed, Figure 6a.

Arrows of different colours were provided in the images of the fractures to identify the various microstructural effects:

(1) Areas without SiC particles—pink arrows;

(2) Voids—orange arrows;

(3) Defragmentation of SiC particles—yellow arrows;

(4) Cracks of SiC particles—blue arrows.

The AA2124 + 17 vol.% SiC with the reinforcing particles of 0.6 µm (specimen 6) was initially subjected to a nominal applied stress of 70 Mpa. It did not reach tertiary creep and the test was stopped after about 530 h. The voids were formed close to each other locally under the loading conditions applied (Figure 7). Single voids did not exhibit a clear tendency to grow. Subsequently, the stress increase of 1 Mpa was applied but the seven specimens did not reach tertiary creep either and the test was stopped after about 1600 h. The stress of 71 Mpa increased the proportion of material defects in the form of merging voids that created material discontinuities (Figure 8).

The specimen 6a images are presented for comparison (Figure 9), after creep carried out at 70 Mpa until fracture was achieved (1300 h). The specimen 6a also did not reach tertiary creep. In this case, more defects were observed than for specimen 6 tested under the same stress of 70 Mpa, but the test was interrupted much earlier, i.e., after about 530 h.

Creep tests at higher stress levels equal to 90 and 91 Mpa were performed on the AA2124 + 25 vol.% SiC composite reinforced by 0.6 µm particles. Morphologically similar voids were observed in the measurement zones of the specimens (Figure 10 and Figure 11). However, it can be noted that the number of voids was greater in specimen AA2124 + 25 vol.% SiC (0.6 μm) subjected to 91 Mpa at 300 °C than in specimen AA2124 + 25 vol.% SiC (0.6 μm) subjected to 90 Mpa at 300 °C.

**Figure 12:**
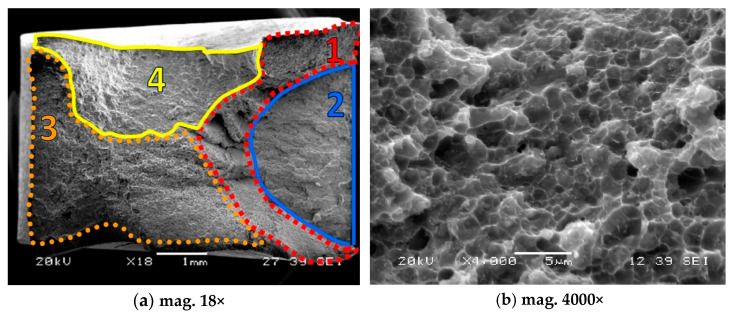
Fracture regions of the composite reinforced by 17 vol.% of SiC, 1—mixed (brittle-plastic) fracture zone for dominant plastic deformation, 2—mixed (brittle-plastic) region for lower contribution of plastic deformation, 3—brittle region, 4—tearing zone (brittle-plastic section).

**Figure 13:**
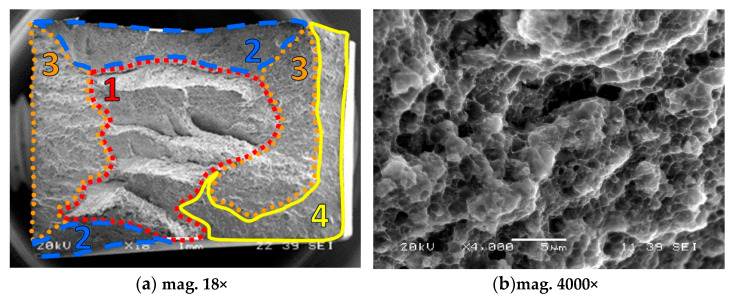
Fracture zones of the composite reinforced by 25 vol.% of SiC: 1—mixed (brittle-plastic) fracture zone for dominant plastic deformation, 2—mixed (brittle-plastic) region for lower contribution of plastic deformation, 3—brittle region, 4—tearing zone (brittle-plastic section).

The fracture sections of the composite specimens reinforced by the 0.6 µm particles are shown in Figure 12 and Figure 13. It is clear that the tested materials have a mixed fractured surface. Numerous dimples are visible on both fracture areas. The voids resulting from the creep process were also observed. Moreover, the coalescence of voids that form cracks is well visible in Figure 12b and Figure 13b.

Quantitative analysis of voids and particle fragmentation is presented in Table 4.

## 4. Conclusions

The analysis of creep tests carried out at 300 °C leads to the conclusion that creep resistance of the AA2124 reinforced by the SiC particles is higher for the materials with finer SiC particles and increases with their content increase. The lowest creep resistance was observed for the AA2124 + 17 vol.% SiC (3 µm) (the range of stress was equal to 50–60 MPa), while the most creep strain resistant was the AA2124 + 25 vol.% SiC (0.6 µm) (stress range was equal to 85–92 MPa). The stress range considered for the AA2124 + 17 vol.% SiC (0.6 µm) was equal to 65–75 MPa. The AA2124 + 25 vol.% SiC (0.6 µm) resisted stresses up to 91 MPa, the AA2124 + 17 vol.% SiC (0.6 µm) up to 71 MPa, while the AA2124 + 17 vol.% SiC (3 µm) up to 55 MPa.

A high creep strain sensitivity of the composites tested was identified in this research for a very narrow range of stress values. It enabled the assessment of a threshold stress range separating a slow and fast creep in the composites tested reflected by low minimum creep rate plus long lifetime and high minimum creep rate plus short lifetime, respectively. In the case of the AA2124 + 17 vol.% SiC (3 µm) such stress range was equal to about 5 MPa, while for the AA2124 + 17 vol.% SiC (0.6 µm) and AA2124 + 25 vol.% SiC (0.6 µm) it was even shorter, limited to around 1 MPa. It means that a very small change in stress applied to the structural element at elevated temperatures may lead to its very rapid collapse or even the whole structure’s destruction.

The results of microstructural and fractographic analysis of the materials tested after creep indicate a diverse character of degradation. In general, the voids appeared as a consequence of the creep process applied. The qualitative metallography of specimens containing 17 vol.% SiC showed that the AA2124 + 17 vol.% SiC (0.6 µm) specimen subjected to 70 MPa at 300 °C was the most damaged (void density equal to 0.00194–1/μm^2^). This is due to the longest operation time of 1300 h. The number of voids in the AA2124 + 25 vol.% SiC (0.6 µm) specimen subjected to 91 MPa at 300 °C seems to be higher (0.00463–1/μm^2^) than in the AA2124 + 25 vol.% SiC (0.6 µm) subjected to 90 MPa at 300 °C (0.00088–1/μm^2^). It was also shown that a greater content of the reinforcement was conducive to the formation of brittle fracture due to the lower plastic strain contribution in the metal matrix of the composite. A fragmentation of SiC occurred as the result of creep process in the composites with coarser reinforcing particles. Moreover, the microscopic images revealed that in both types of composites reinforced by SiC of 0.6 µm and 3 µm, only small areas without the reinforcement were observed.

This research can be treated as the first attempt for a better understanding of the visco-plastic behaviour of aluminium alloy-based composites reinforced by the SiC particles. More extensive experimental and numerical analyses are required to better reflect complex creep behaviour of the composites under conditions indicated in this experimental programme. The strengths of the studies are expressed by the unique creep data and microstructural observations after prior creep deformation of the materials tested.

## Figures and Tables

**Table 1:** Tests details for AA2124 + 17 vol.% SiC (3 µm).

Specimen	Temperature [°C]	Stress [MPa]	Test Time [h]	Strain [%]	Minimum Creep Rate [s^−1^]	Test End Notes
Specimen 1	300	50	1180	0.65	6.5 × 10^−10^	Test stopped
Specimen 2	300	55	195	1.5	1.4 × 10^−8^	Test stopped
Specimen 3	300	55	198	1.7	1.3 × 10^−8^	Test stopped
Specimen 4	300	39/60	(118 + 3)	2	1.2 × 10^−6^ (60 MPa)	Test stopped

**Table 2:** Tests details for AA2124 + 17 vol.% SiC (0.6 µm).

Specimen No.	Temperature [°C]	Stress [MPa]	Test Time [h]	Strain [%]	Minimum Creep Rate [s^−1^]	Test End Notes
5	300	65	479	0.44	5.3 × 10^−11^	Test stopped
6	300	70	1645	0.9	3.5 × 10^−10^	Until rupture
6a	300	70	530	0.78	5.3 × 10^−10^	Test stopped
7	300	71	1591	1.16	6.9 × 10^−10^	Until rupture
8	300	72	0.4	2	5.3 × 10^−6^	Test stopped
9	300	75	0.4	26	-	Test stopped
10	300	75	0.2	2	-	Test stopped

**Table 3:** Tests details for AA2124 + 25 vol.% SiC (0.6 µm).

Specimen No.	Temperature [°C]	Stress [MPa]	Test Time [h]	Strain [%]	Minimum Creep Rate [s^−1^]	Test End Notes
11	300	85	1100	0.76	6.4 × 10^−10^	Until rupture
12	300	90	470	0.76	1.3 × 10^−9^	Until rupture
13	300	91	649	1.04	1.3 × 10^−9^	Until rupture
13a	300	91	624	0.8	7.5 × 10^−10^	Test stopped
14	300	92	0.096	1.89	-	Test stopped

**Table 4:** Quantitative analysis of defects.

Figures Number	The Size of Reinforcement	Measurement Conditions	Void Density N [1/μm^2^]	Volume Fraction of Voids V_V_ [%]	Mean Feret Diameter of Void F_śr_ [μm]	Particle Fragmentation P [1/μm^2^]
Figure 5.	3 μm AA2124 + 17 vol.% SiC specimen 1	300 °C 50 MPa	0.00100	0.41664	2.08333	0.00031
Figure 6.	3 μm AA2124 + 17 vol.% SiC specimen 3	300 °C 55 MPa	0.00150	0.55556	1.46755	0.00056
Figure 7.	0.6 μm AA2124 + 17 vol.% SiC specimen 6	300 °C 70 MPa	0.00088	0.69444	2.35294	-
Figure 8.	0.6 μm AA2124 + 17 vol.% SiC specimen 7	300 °C 71 MPa	0.00106	0.90278	3.53992	-
Figure 9.	0.6 μm AA2124 + 17 vol.% SiC specimen 6a	300 °C 70 MPa	0.00194	0.69444	2.66386	-
Figure 10.	0.6 μm AA2124 + 25 vol.% SiC specimen 4	300 °C 90 MPa	0.00088	0.45139	2.50000	-
Figure 11.	0.6 μm AA2124 + 25 vol.% SiC specimen 13	300 °C 91 MPa	0.00463	0.52083	2.82250	-

## Data Availability

The original contributions presented in the study are included in the article. Further inquiries can be directed to the corresponding author.
